# Detection of Nitazenes (2-Benzylbenzimidazoles)—Novel Synthetic Opioids in Coronial Casework in Victoria, Australia

**DOI:** 10.3390/metabo16060358

**Published:** 2026-05-26

**Authors:** Lachlan Scully, Jared W. Castle, Matthew Di Rago, Hans H. de Boer, Jennifer Schumann, Kerryn Crump, Linda Glowacki, Dimitri Gerostamoulos

**Affiliations:** 1 Victorian Institute of Forensic Medicine, Southbank, VIC 3006, Australia; jared.castle@vifm.org (J.W.C.); matthew.dirago@vifm.org (M.D.R.); hans.de.boer@vifm.org (H.H.d.B.); jennifer.schumann@vifm.org (J.S.); kerryn.crump@vifm.org (K.C.); linda.glowacki@vifm.org (L.G.); dimitri.gerostamoulos@vifm.org (D.G.); 2 Department of Forensic Medicine, Monash University, Melbourne, VIC 3006, Australia

**Keywords:** nitazenes, 2-benzylbenzimidazole, novel synthetic opioids, fatalities, overdose, novel psychoactive substance

## Abstract

**Background**: The 2-benzylbenzimidazoles or nitazenes are an evolving class of highly potent mu-opioid receptor agonists. Nitazenes were originally developed in the late 1950s for pharmaceutical use as analgesics; however, due to their extreme potency and the risk of adverse health outcomes, pharmaceutical research was discontinued. Since 2019, nitazenes have emerged as illicit drugs of abuse, causing significant concern. From 2021, they have been detected in both coronial and clinical casework in Victoria, Australia. This study examined nitazene-related coronial casework in Victoria from 2021 to 2025 to explore the trends and characteristics of nitazene-related deaths. **Methods**: Relevant cases were identified from the Victorian Institute of Forensic Medicine’s (VIFM’s) case management system. Data were collated and analysed from all coronial cases where a nitazene was detected by a toxicological analysis between 1 January 2021 and 31 December 2025. Trend comparisons were made with nitazene detections reported in other countries. **Results**: Nitazenes were detected in 23 deaths from a total of approximately 33,108 coronial cases admitted to the VIFM for investigation over the time period. The age range was 17–45 years, with a median of 32 and with 87% of the deaths being male. The nitazenes detected were protonitazene (*n* = 14), metonitazene (*n* = 5), isotonitazene (*n* = 2), *N*-pyrrolidino etonitazene (*n* = 2), *N*-desethyl isotonitazene (*n* = 1), methylenedioxynitazene (*n* = 1) and etodesnitazene (*n* = 1). Two cases contained more than one nitazene; both involved protonitazene, one involved metonitazene, and the other involved *N*-desethyl isotonitazene and methylenedioxynitazene. The timeline of detection of these nitazenes displays similarities with emergence trends in other countries. The nitazene concentrations ranged from 0.1 to 33 ng/mL. Broad polydrug usage was evident in all cases, with other drugs co-detected in the blood including stimulants (particularly, methylamphetamine (48%) and cocaine (44%)) as well as pharmaceutical benzodiazepines (43%) and pharmaceutical opioids (22%), and 13% had 6-monoacetylmorphine detected in either blood or urine. Novel benzodiazepines (39%) were also common, including bromazolam, which was co-detected in 35% of cases. Nineteen deaths were attributed solely to nitazene-related mixed-drug toxicity, while the remaining four cases were attributed to cardiac- and pulmonary-related disease, with polydrug use deemed a contributing factor. **Conclusions**: This novel case series adds comprehensive toxicological information to the body of evidence reinforcing the high risk of harm associated with the use of nitazenes. It is imperative that toxicology services continue to monitor for nitazenes to promote community awareness against nitazene-related harm.

## 1. Introduction

The emergence and rapid evolution of novel synthetic opioids (NSOs) represents a critical and growing global public health concern. In the United States (USA) from 1999 to 2018, the number of drug overdose deaths involving synthetic opioids other than methadone increased per 100,000 persons from 0.3 to 9.9 [1]. This was largely driven by fentanyl and its analogues, in what has become known as the USA opioid crisis, with deaths attributed to synthetic opioids rising from 14.9% to 45.9% of all opioid-related fatalities between 2010 and 2016 [2,3]. Class-wide bans on fentanyl analogues in the USA and China have been associated with the emergence of non-fentanyl NSOs on illicit drug markets [4,5,6]. To date, these NSOs have demonstrated a high toxicity and have contributed to the ongoing escalation of opioid-related harms associated with use of synthetic opioid compounds [7]. These structurally diverse compounds most notably include U-series compounds, such as naphthyl U-47700, and the nitazenes (2 benzylbenzimidazoles), which are currently the most prevalent class of non-fentanyl-based NSO globally [1,5,8,9].

**Figure 1:**
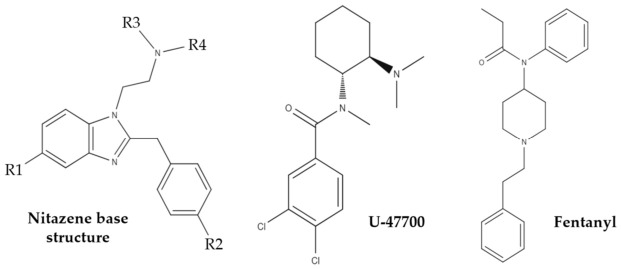
Base nitazene benzimidazole core structure with four variable (R1–R4) regions compared to structurally unrelated synthetic opioids U-47700 and fentanyl.

Nitazenes were first synthesised in the late 1950s by the Swiss pharmaceutical company Chemische Industrie Basel in search of a safer analgesic alternative to morphine [10,11,12]. Structurally, they consist of a benzimidazole core with a branching nitrite (R1) group, *N*-ethylamine side chain (R2) and phenylalkyl chain (R3) (Figure 1, Table 1) [9,13]. Like other synthetic opioids, these compounds are potent mu-opioid receptor agonists that cause desirable sensations such as analgesia, euphoria and relaxation, but also the risk of adverse effects, including fatigue, sleepiness, confusion, vomiting and nausea [9,14,15]. *In vitro* characterisation studies have investigated the pharmacological activity of a range of nitazenes, estimating potencies both greater than and less than fentanyl, with the strongest (*N*-pyrrolidino etonitazene) being approximately 40 times greater [11,13,16]. Their use is associated with a substantial risk of an opioid toxidrome, including potentially fatal dose-dependent respiratory depression. Consequently, these compounds have never been approved for clinical use [5,9,13].

Nitazenes first emerged on the illicit drug market in 2019, with several analogues sold online and discussed in drug-use forums, as well as detections in drug-use materials [12]. Isotonitazene was the first nitazene reported in a toxicology casework sample in Canada in March 2019, with subsequent detections following in Europe and the USA in July 2019 [17,18,19]. From mid-to-late 2019 to mid-2020, dozens of intoxications and fatalities involving isotonitazene were reported in the USA and across several European countries, leading to its scheduling in the USA in August 2020 [5]. This resulted in a sharp reduction in isotonitazene use and coincided with the emergence of several new nitazene analogues, including dozens of fatal and nonfatal intoxications involving metonitazene from November 2020 onwards [5]. Butonitazene, etodesnitazene, *N*-pyrrolidino etonitazene, etonitazepipne, flunitazene, metodesnitazene, and protonitazene emerged in 2021 and 2022, and were associated with both intoxications and fatalities [5,14,20,21,22]. The autopsy findings reflected typical, but non-specific, observations following an opioid overdose, including pulmonary congestion/a high lung weight, cerebral oedema, a full bladder and gastric contents in the airways [5,15]. Nitazenes have been detected in powders, tablets and within e-liquids, with diverse routes of administration including intravenous, intranasal, rectal and vaporisation [5,23,24,25].

By 2023, nitazenes accounted for 43% of newly detected NSOs globally, reflecting a significant shift in the composition of illicit opioid markets since the emergence of fentanyl [26]. The rapid proliferation of new nitazene analogues is driven by the structural flexibility of the central benzimidazole core, which permits extensive chemical modification and a wide range of structural analogues. Indeed, since 2022, methylenedioxynitazene, *N*-desethyl isotonitazene, *N*-desethyl etotonitazene, *N*-desethyl metonitazene, *N*-desethyl protonitazene, *N*-pyrrolidino isotonitazene, *N*-pyrrolidino protonitazene, *N*-pyrrolidino metonitazene and 5-methyl etodesnitazene have all been reported as new analogues in the USA [9,27,28] (Table 1). The continued emergence of nitazenes poses a significant challenge to existing legislation. Notably, many identified nitazene analogues were not described in the original patent literature from the late 1950s, suggesting ongoing clandestine synthesis of previously unreported analogues. The rapid emergence of nitazenes, coupled with limited clinical understanding of nitazene-related overdoses, presents significant challenges for medico-legal practitioners [5]. Improved knowledge and information sharing are critical to addressing this ongoing threat of nitazenes.

The emergence of nitazenes in Australia has raised significant public health concerns. Various nitazenes have been detected in multiple Australian jurisdictions as part of the Emerging Drug Network of Australia study, which monitors illicit drug presentations in major hospital emergency departments [14,29,30]. Victorian identifications have consequently led to the release of several public health warnings regarding the circulation of nitazenes in the illicit drug supply [31,32,33]. This study reports the detection of nitazenes in coronial casework in the state of Victoria from January 2021 to December 2025, with post-mortem concentrations and key forensic toxicological findings not previously reported, adding to our understanding of the toxicity of these emerging compounds in our community.

## 2. Materials and Methods

### 2.1. Chemicals and Standards

The reagents used for sample preparation were a Tris(hydroxyl)aminomethane base (Sigma-Aldrich, Sydney, Australia), *n*-butyl chloride and isopropanol (Merck, Darmstadt, Germany). Liquid-chromatography mobile phases were prepared using ammonium formate (Sigma-Aldrich, Sydney, Australia), formic acid, acetonitrile and methanol (all sourced from Merck, Darmstadt, Germany). All other chemicals were of analytical grade. The water was ultrapure deionized water made using a Milli-Q Direct 8 system (Merck-Millipore, Melbourne, Australia). All nitazene drug standards were sourced from Cayman Chemical (Ann Arbor, MI, USA), and fentanyl-d5 was purchased from Novachem (Melbourne, Australia). All analyte calibrators and working solutions were prepared in methanol.

### 2.2. Setting and Pathology

The Victorian Institute of Forensic Medicine (VIFM) is an independent statutory authority located in Melbourne, Australia, serving a population of approximately seven million people. Approximately 7500 medicolegal death investigations are conducted annually on behalf of the Victorian Coroner’s Court. The Coroner’s Court investigates prescribed categories of reportable deaths, including those that are sudden, unexpected, violent or unnatural.

Following admission to the mortuary, each body was subject to a full-body postmortem CT scan (Siemens, Munich, Germany), collection of a femoral blood sample, and an external examination. Based on case circumstances, the external examination and radiological findings, a forensic pathologist recommended to the Victorian coroner whether further examination was warranted, such as an autopsy and specimen collection for auxillary tests such as toxicology and biochemistry. Sixty-one percent of cases in this series underwent a full autopsy, which always included histology of all major organs. In the remainder, a full-body postmortem CT scan, an external examination and a toxicological analysis were deemed sufficient. Femoral blood specimens were available for all cases included in this study.

### 2.3. Collection of Specimens

Femoral blood was collected as soon as possible following body admission to the mortuary at the VIFM as part of the routine case triage process. It was collected by puncture to the femoral vein into 10 mL polypropylene tubes containing 1% (*w*/*v*) sodium fluoride/potassium oxalate (Sarstedt, Adelaide, Australia). These admission samples with a preservative were stored at +4 °C before transfer to the toxicology laboratory as soon as practicable (typically within 24 h of collection), where they were also stored at +4 °C.

Decedents were stored in dedicated cool rooms at +4 °C. If an autopsy was performed, two additional femoral blood samples with a preservative were collected. Both additional preserved blood samples were also stored at +4 °C before transfer to the toxicology laboratory as soon as practicable (also typically within 24 h of collection), where one was stored at +4 °C and the other at −20 °C.

One death occurred in hospital and had antemortem specimens available. The earliest specimen collected closest to the hospital admission was analysed. Antemortem specimens were also stored at −20 °C until the analysis.

### 2.4. Toxicological Analysis

The drug analysis of case blood samples consisted of an alkaline liquid–liquid extraction followed by an LC–MS/MS screen using a Shimadzu Prominence HPLC (Kyoto, Japan) and SCIEX 3200 QTRAP MS-MS (Marlborough, MA, USA) for 327 common medicines and illicit substances [34]. Where the circumstances and results suggested the possibility of novel psychoactive substances (NPSs), an NPS-specific LC–MS/MS screen using a Shimadzu Nexera UHPLC (Kyoto, Japan) and SCIEX QTRAP 4500 (Marlborough, MA, USA) targeting over 550 NPSs, including novel opioids, benzodiazepines, stimulants, hallucinogens and synthetic cannabinoids, was performed. New NPSs are routinely added to this method, and subsequently, the breadth of NPSs and nitazene analogues targeted using this method has changed over time (Table 2). The cases were also analysed using an Agilent 1290 Infinity II UHPLC and 6546 QTOF-MS (Santa Clara, CA, USA) (LC-QTOF/MS) operating in the All Ions and Auto-MS/MS modes, in a “suspect screening” approach utilizing the HighResNPS.com database, and substances were added as “suspect targets” from the literature periodically following updates to this database [35]. Significant NPS-specific LC-MS/MS or LC-QTOF-MS detections were then confirmed and quantitated using LC-MS/MS. Detailed chromatographic conditions and MS parameters for the NPS-specific LC-MS/MS and LC-QTOF-MS methods were as described previously [35].

All nitazene detections were confirmed against certified reference material and quantitated using LC-MS/MS. However, due to the extended timeframe over which positive cases were identified and delays in availability of reference material, there were variations in the confirmation techniques. For each confirmation, individual calibration curves of 8–10 points were constructed for each analyte by spiking 10 μL of working calibrant solution into 100 μL of drug-free blood. For most analyses, the calibration range was 0.05–50 ng/mL, although a lower upper limit of 20 or 30 ng/mL was applied in some instances when the expected analyte concentration was lower following an initial result obtained via the NPS-specific LC–MS/MS screen. The calibration model was linear through zero, affording standard accuracies within ±20% and r > 0.99. The limit of detection was not systematically investigated, but was at least 0.05 ng/mL for all nitazenes targeted (signal-to-noise greater than 3). Quality controls (QCs) were prepared by a secondary analyst in drug-free blood from diluted drug solutions with concentrations between 3 and 20 ng/mL, and were used to monitor the method accuracy and precision. The QC concentrations were considered acceptable within 30% of the nominal spiked concentration. The liquid–liquid extraction was the same as applied in the laboratory’s basic-neutral drug screen, consisting of 0.1 mL of blood buffered with 0.2 mL of 2 M Trizma buffer and extracted in 1 mL of butyl chloride:isopropanol (9:1) [34]. The LC-MS/MS conditions for the NPS-specific LC-MS/MS have been published previously, with injection volumes ranging from 10 to 40 µL depending on instrument availability and performance [35]. Separation was conducted using a SCIEX Exion (Marlborough, MA, USA) or Shimadzu Nexera (Kyoto, Japan) UHPLC with confirmation with a SCIEX QTRAP 4500 MS/MS. Analyte identification was confirmed if the retention times were within 2% and the MRM ratios within 20% of the matrix-matched reference material.

As part of routine toxicology testing, an ethanol and simple volatile analysis was also performed for all cases using headspace gas chromatography with flame ionisation detection. Screening for 157 acidic and neutral drugs and poisons, a hair analysis for 63 drugs of abuse and metabolites using LC-MS/MS, and a gamma-hydroxybutyrate (GHB) analysis using validated in-house GC/MS and LC-MS/MS methods were performed depending on the case circumstances and results of the initial toxicological testing.

### 2.5. Case Review

There were 21,486 coronial cases that underwent minimum blood toxicology testing between January 2021 and December 2025. Of these, 1892 underwent further testing using the NPS-specific LC-MS/MS screen. Cases where a nitazene was detected were collated, and the demographic and key toxicological findings were extracted and summarised.

## 3. Results

### 3.1. Cohort Demographics and Causes of Death

From Q1 (quarter 1) 2021 to Q4 2025, there were 23 nitazene-related fatalities. Most cases were male (87%), with an age range of 17–45 years and a median of 32 years. Most were 36–45 years of age (*n* = 9, 39%), followed by 26–35 years (*n* = 8, 35%) and 16–25 years (*n* = 6, 26%). Death was predominantly deemed to be due to mixed-drug toxicity (*n* = 19, 82%), with the remaining 18% attributed to cardiac or pulmonary disease with polydrug use deemed a contributing factor (*n* = 4, 18%). Only one case had presented to the hospital prior to death.

### 3.2. First Detections

**Figure 2:**
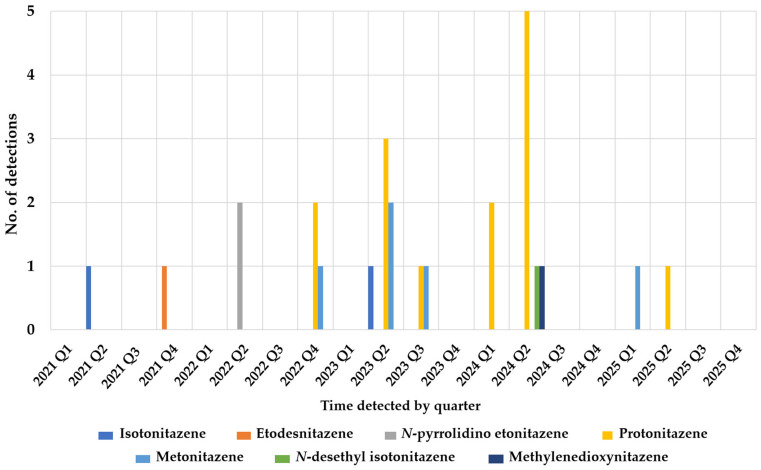
Quarterly nitazene detections in coronial forensic casework at the VIFM from January 2021 to December 2025.

In total, seven different nitazenes were identified (Table 3—structures shown in Table 1), with isotonitazene first detected in Q1 2021. Initial detections of the remaining six nitazenes ranged between Q4 2021 (etodesnitazene) and Q2 2024 (*N*-desethyl isotonitazene and methylenedioxynitazene) (Figure 2). The majority of detections were identified using the NPS-specific LC-MS/MS method, with the exception of etodesnitazene, which was identified using LC-QTOF-MS in Q4 2021 via the HighResNPS library. Methylenedioxynitazene was detected atypically during a retrospective analysis for other research purposes after addition to the NPS-specific LC-MS/MS method in September 2025.

### 3.3. Casework Detections and Trends

The concentrations detected ranged from 0.1 to 33 ng/mL (Table 3). *N*-Pyrrolidino etonitazene had the lowest mean concentration of 0.4 ng/mL. Of the analogues that were detected more than once, metonitazene was detected with the highest average concentration of 15.7 ng/mL (five cases). Protonitazene was the most abundant nitazene appearing in 61% of cases (14 cases) with an average concentration of 2.1 ng/mL (range: 0.1–11.6 ng/mL), while metonitazene was the next most common, detected in 22% of cases. Protonitazene and metonitazene were detected from Q4 2022, with 13 of the 18 deaths containing these compounds occurring during 2023 and 2024. All other analogues were detected twice or less, with *N*-pyrrolidino etonitazene both occurring in Q2 2022, while the two isotonitazene detections occurred about 3 years apart, between Q1 2021 and Q2 2024. More than one nitazene analogue was detected in two cases that both involved protonitazene; one involved metonitazene and the other involved *N*-desethyl isotonitazene and methylenedioxynitazene (see Figure 2).

### 3.4. Co-Detections

A summary of all drug detections is provided in Table 4. All cases except one were found to have at least one other drug present in the blood. Stimulants were the most common drug class co-detected (78% of cases), with methylamphetamine being the most prevalent (48%), followed by cocaine and its metabolites (44%) and 3,4-methylenedioxy-N-methamphetamine (MDMA) (13%). Pharmaceutical benzodiazepines were also detected in 43% of cases, with diazepam or nordiazepam being the most prevalent (30%), followed by alprazolam (13%), with 35% of cases containing at least one stimulant and a pharmaceutical benzodiazepine. At least one additional non-nitazene NPS was detected in 39% of the total cases, each containing at least one novel benzodiazepine. Bromazolam was the most prevalent (89%) in this subset of cases containing a non-nitazene NPS, followed by desalkylgidazepam (40%) and clonazolam (22%). Synthetic cathinones (13%) were also co-detected, with *N,N*-dimethylpentylone (and the metabolite pentylone) being the most common, in 9%. Pharmaceutical opioids (22%) and cannabinoids (17%) were also present, along with ethanol (17%) and GHB in blood or urine (13%). 6-Monoacetylmorphine was detected in three cases (13%) (two in urine).

## 4. Discussion

This case series highlights the emergence of nitazenes in Victoria, Australia, and their significance as a growing public health threat. Previous case series have noted a typical nitazene user as a middle-aged male between 30 and 50 years old [5,20,23,36,37]. The mean age of 32 years within our series, as well as the high representation of males (87%), is consistent with these trends [38].

Currently, there is limited understanding of what constitutes toxic and/or lethal concentrations of nitazenes. Nitazenes in biological fluids are typically detected at low to sub-ng/mL levels. This case series contained concentrations of less than 33 ng/mL and reflects previously reported concentrations in nitazene-related deaths of up to 40 ng/mL [5,10,18,37,39]. For individual analogues detected in this series, the concentrations also largely reflect those previously published for similar related fatalities other than methylenedioxynitazene, of which concentrations had not been reported in the literature prior to this study [28]. Isotonitazene has been reported in postmortem casework with average concentrations of 2.3 [40], 2.2 [36] and 1.2 ng/mL [18], and the average protonitazene concentration in one publication was 6.1 ng/mL [41], in comparison to our mean of 2.1 ng/mL. Previous series reported individual metonitazene concentrations of 0.10, 0.49 and 1.5 ng/mL [40], and an average of 6.3 ng/mL from 20 cases [20]. While lower than the mean concentration observed in our casework (15.7 ng/mL), these concentrations still fall within the range found in our five positive cases (0.4–33 ng/mL).

Our small study size, along with very few detections of certain analogues, is a significant factor limiting interpretation. It is clear, however, that the relatively low concentrations observed in this cohort are suggestive of strong pharmacological effects. Previous case series, as well as the reported potencies of various nitazenes *in vitro*, support this, as does the fact that only 1/23 cases made it to the hospital prior to death [13]. A comparison of individual analogue concentrations in this series to established *in vitro* potencies revealed that analogues with a greater relative estimated potency are correlated with lower average concentrations detected [13,21]. These are valuable insights, as, outside of a limited number of NPSs for which some pharmacokinetics are understood, typically a paucity of known pharmacokinetics limits interpretation of any measured concentrations of NPSs with any level of confidence [42].

Furthermore, the stability of nitazenes *in vitro* in whole-blood specimens is not well studied. One study by Walton et al. found eight different nitazenes (isotonitazene, protonitazene, etonitazene, metonitazene, clonitazene, flunitazene, *N*-desethyl isotonitazene, and 4′-hydroxy nitazene) to be stable in blood stored at +4 °C over a 60-day time period [12], although it was not clear if a preservative was used. Recently, it has been found that metonitazene may degrade to 5-aminometonitazene and then 5-acetamidometonitazene in unpreserved post-mortem specimens, highlighting a vulnerability of the nitro group at position 5 of the benzimidazole ring to enzymatic degradation [43]. It is reasonable to suspect that nitazenes possessing a nitro group, the “nitronitazenes”, such as the *N*-pyrrolidino etonitazene, *N*-desethyl isotonitazene, protonitazene, and isotonitazene reported in this case series, may undergo degradation prior to the collection of blood in preserved specimen tubes to form analogous amino and acetamido degradation products [43]. Additionally, previous studies comparing peripheral and cardiac blood in deaths involving metonitazene, flunitazene and *N*-pyrrolidino protonitazene revealed central/peripheral (C/P) ratios of 2.4, 2.3 and 4, respectively [11,20]. C/P distributions were also investigated in several deaths involving isotonitazene, noting C/P ratios of 1.5, 0.7, 1.9 and 0.9 [18,44,45]. Despite variation in the C/P ratios, these data highlight the prospect that nitazenes, and particularly the “nitronitazenes”, may exhibit a tendency for post-mortem redistribution [46]. Chen et al. identified three “nitronitazenes”: metonitazene, *N*-desethyl isotonitazene and *N*-pyrrolidino etonitazene. Each degraded in postmortem femoral blood collected from rats over a 7-day period, with only 14% of the nitazenes detected in day 1 samples remaining by day 7 in samples stored in the fridge for one month [47]. They purported that nitazene deaths may therefore be underestimated by up to a third. In our study, only the amino degradant for isotonitazene (5-aminoisotonitazene) was included in the NPS-specific LC-MS/MS screen at the time of analysis. Due to these stability concerns, it is possible that not all cases where nitronitazenes were used prior to death have been captured in this case series.

Similar to previous case series of nitazene-related fatalities, substantial polydrug use limited understanding of the nitazene contribution to fatal drug toxicity [5]. All cases except one had analytical evidence of polydrug abuse, with only *N*-pyrrolidino etonitazene (0.6 ng/mL) detected in this case that underwent an autopsy. Across the cohort, the high prevalence of co-detected stimulants and pharmaceutical and novel benzodiazepines, most commonly bromazolam, is concerning and reflects trends in other reported nitazene-related deaths [5,18,29,36,37]. Benzodiazepines are central nervous system (CNS) depressants with sedative effects that have a synergistic effect when taken in combination with other CNS depressants, such as opioids and particularly heroin and GHB, both of which were detected in 13% of the cases [48]. There are significantly higher risks of respiratory depression and death, exacerbated with the use of combinations of novel benzodiazepines, heroin, GHB and NSOs [48]. The detection of xylazine in one case in the series is significant in this context, as this veterinary anaesthetic has become increasingly prevalent and has exacerbated opioid-related harm by potentiating the harmful effects of other opioids, but also through its own toxicity [49]. It is well known that NPSs can contain widely different constituents to that expected by the user, which may result in a greater likelihood of unpredictable outcomes or an overdose [50]. It is noteworthy that, for the four cases with desalkylgidazepam detected, its detection may have been an artefact of bromazolam, as three-quarters of these detections occurred alongside bromazolam. Although its presence as an artefact has not yet been discussed extensively in the literature, the co-detection of desalkylgidazepam and bromazolam was reported by Mérette et al. in a series of 74 post-mortem blood samples positive for desalkylgidazepam in British Columbia, Canada, in 2022, where 47.6% were co-detected with bromazolam [51]. It is also worth noting that four deaths in this study occurred together, with cocaine co-detected with protonitazene in the blood of all decedents in this incident in what was reportedly unexpected exposure to nitazenes. This trend is largely evident across the study, with all cases in our cohort listing mixed-drug toxicity as either a sole (82%) or partial (18%) cause of death in the setting of other cardiac or pulmonary disease, highlighting polydrug use as a public health concern.

The use of nitazenes with other illicit drugs emphasises the importance of the availability of naloxone at a community level, as studies thus far have highlighted that standard parenteral doses are effective, although repeated dosages may be required [52,53,54,55]. The availability of drug-checking services to support safer drug-use behaviours, as well as timely communication of urgent public health warnings when such compounds are circulating within the community, are also important initiatives to prevent future nitazene-related harm.

The increase in nitazene-related deaths reported in this case series shows similarity to trends in the USA and Europe. In our cohort, six and four detections of protonitazene and metonitazene, respectively, occurred between Q4 2022 and Q3 2023. This coincided with increased protonitazene and metonitazene detections in the USA during approximately the same period, as reported by The Center for Forensic Science Research and Education [27]. Similarly, two detections of *N*-pyrrolidino etonitazene in this series observed during Q1–Q2 2022 were also correlated with a spike in detections simultaneously observed in the USA [21,27]. These relative associations may suggest production and circulation of these nitazenes at the same time. However, the sole detection of *N*-desethyl isotonitazene in our series occurred approximately two years after its emergence in Europe and the USA (2022) [25,37,56,57]. *N*-desethyl isotonitazene is a metabolite of isotonitazene, but has greater in vivo antagonism of the mu-opioid receptor and has been detected in intoxications and fatalities in isolation [13,23,37]. This compound was detected in the absence of isotonitazene in a single case in our study, which suggests that it may have been taken directly, rather than detected via isotonitazene metabolism.

A limitation of this study is that not all cases submitted to the VIFM are necessarily tested for nitazenes. Specific testing for NPSs, including nitazenes, is performed based on the case circumstances, drug-use history, preliminary drug testing or postmortem findings. Although the threshold for testing is considered relatively low, it is possible that some nitazene-related deaths during the study period may have remained unidentified.

## 5. Conclusions

The emergence of nitazenes remains a significant public health concern, both locally and globally. Fatalities involving nitazenes have risen, with new nitazene analogues emerging in the unregulated drug supply. The deaths reported in this series that were attributed to nitazene-related mixed-drug toxicity adds to a concerning trend of nitazene use in Australia and their profound toxicity, particularly in the setting of polydrug use, and even at low concentrations. It is imperative that toxicology laboratories, public health officials, and policy makers remain vigilant and adaptable in their approaches to public safety and awareness regarding the continued emergence of potent NSOs.

## Figures and Tables

**Table 1:** Variable region structures of nitazenes detected in forensic samples and drug material since 2019.

	R1	R2	R3	R4
Butonitazene	NO_2_	O(CH_2_)_3_CH_3_	CH_2_CH_3_	CH_2_CH_3_
Etodesnitazene	H	OCH_2_CH_3_	CH_2_CH_3_	CH_2_CH_3_
Etonitazene	NO_2_	OCH_2_CH_3_	CH_2_CH_3_	CH_2_CH_3_
Flunitazene	NO_2_	F	CH_2_CH_3_	CH_2_CH_3_
Isotonitazene	NO_2_	OCH(CH_3_)_2_	CH_2_CH_3_	CH_2_CH_3_
Methylenedioxynitazene	NO_2_	OCH_2_O	CH_2_CH_3_	CH_2_CH_3_
Metonitazene	NO_2_	OCH_3_	CH_2_CH_3_	CH_2_CH_3_
Metodesnitazene	H	OCH_3_	CH_2_CH_3_	CH_2_CH_3_
*N*-Desethyl isotonitazene	NO_2_	OCH(CH_3_)_2_	H	CH_2_CH_3_
*N*-Desethyl etonitazene	NO_2_	OCH_2_CH_3_	H	CH_2_CH_3_
*N*-Desethyl protonitazene	NO_2_	O(CH_2_)_2_CH_3_	H	CH_2_CH_3_
*N*-Piperidinyl etonitazene	NO_2_	OCH_2_CH_3_	(C_5_H_10_)	-
*N*-Pyrrolidino isotonitazene	NO_2_	OCH(CH_3_)_2_	(C_4_H_8_)	-
*N*-Pyrrolidino protonitazene	NO_2_	O(CH_2_)_2_CH_3_	(C_4_H_8_)	-
*N*-Pyrrolidino etonitazene	NO_2_	OCH_2_CH_3_	(C_4_H_8_)	-
*N*-Pyrrolidino metodesnitazene	-	OCH_3_	(C_4_H_8_)	-
Protonitazene	NO_2_	O(CH_2_)_2_CH_3_	CH_2_CH_3_	CH_2_CH_3_
5-Methyl etodesnitazene	CH_3_	OCH_2_CH_3_	CH_2_CH_3_	CH_2_CH_3_

**Table 2:** Nitazene analogues incorporated into the targeted laboratory NPS screen as of 31 December 2025.

Analogue	Date Incorporated into Novel Psychoactive Substance Screen
Isotonitazene	24 August 2020
Etonitazene	27 August 2020
Clonitazene	11 March 2021
Metonitazene	11 March 2021
Metodesnitazene	28 September 2021
Butonitazene	22 November 2021
Etodesnitazene	22 November 2021
Flunitazene	22 November 2021
Isotodesnitazene	10 December 2021
Protonitazene	10 December 2021
*N*-Pyrrolidino etonitazene	13 January 2022
5-Aminoisotonitazene	23 January 2024
*N*-Desethyl isotonitazene	23 January 2024
*N*-Piperidinyl etonitazene	23 January 2024
sec-Butonitazene	23 January 2024
5-Methyl etodesnitazene	20 February 2024
Ethyleneoxynitazene	1 October 2024
*N*-Desethyl etonitazene	1 October 2024
*N*-Pyrrolidino isotonitazene	1 October 2024
*N*-Pyrrolidino protonitazene	1 October 2024
*N*-Pyrrolidino metonitazene	10 February 2025
3′-Methoxy metodesnitazene	2 September 2025
5-Methyl metodesnitazene	2 September 2025
Menitazene	2 September 2025
*N*-Desethyl protonitazene	2 September 2025
Nitazene	2 September 2025
*N*-Piperidinyl isotonitazene	2 September 2025
*N*-Piperidinyl isotonitazene	2 September 2025
*N*-Piperidinyl metonitazene	2 September 2025
*N*-Piperidinyl protonitazene	2 September 2025
*N*-Pyrrolidino etodesnitazene	2 September 2025
*N*-Pyrrolidino metodesnitazene	2 September 2025
Propylnitazene	2 September 2025
Protodesnitazene	2 September 2025
Methylenedioxynitazene	16 September 2025

**Table 3:** Number of detections (*n*), mean, median, range of concentrations (ng/mL) and date first detected for different nitazenes identified in coronial forensic casework at the VIFM between January 2021 and December 2025, with the estimated potency compared to fentanyl.

Nitazene(s) Detected	*n*	Mean (ng/mL)	Median (ng/mL)	Range (ng/mL)	Date First Detected	Potency Relative to Fentanyl [13,16,21]
*N*-Pyrrolidino etonitazene	2	0.4	0.45	0.3–0.6	Q2 2022	~43×
*N*-Desethyl isotonitazene	1	4.9	4.9	4.9	Q2 2024	~23×
Isotonitazene	2	1.7	1.7	0.1–3.4	Q2 2021	~9×
Protonitazene	14	2.1	1.3	0.1–11.6	Q4 2022	~3.5×
Metonitazene	5	15.7	14.7	0.4–33.0	Q4 2022	~2×
Etodesnitazene	1	32.2	32.2	32.2	Q4 2021	~1/4–1/2×
Methylenedioxynitazene	1	3.7	3.7	3.7	Q2 2024	~1/5×

**Table 4:** Femoral blood concentrations of drugs, causes of death, date of detection and age of decedent in the 23 cases involving nitazenes detected at the VIFM in Victoria, Australia. Note: detections noted as “DET” were detected above the limit of quantitation and not accurately quantified at the time of analysis. The upper limit of quantitation was determined depending on the instrumentation available for use and the specific confirmation/quantitation analysis performed. DET = detected.

Case No.	Nitazene(s) Detected (ng/mL)	Date	Age Category	Other Drugs Detected in Blood (mg/L Unless Specified)	Cause of Death
1	Isotonitazene (3.4)	Q2 2021	30–39	Xylazine 0.5 (ng/mL) Nordiazepam 0.03 Desmethylvenlafaxine 1 Promethazine 0.1	Mixed-drug toxicity
2	Etodesnitazene (32.1)	Q4 2021	30–39	Diazepam 0.4	Mixed-drug toxicity with valvular heart disease
3	*N*-Pyrrolidino etonitazene (0.6)	Q2 2022	<20		Drug toxicity
4	*N*-Pyrrolidino etonitazene (0.3)	Q2 2022	20–29	Flualprazolam 4 (ng/mL) 8-Aminoclonazolam 5 (ng/mL) Etizolam 8 (ng/mL) Flubromazepam 32 (ng/mL) Bromazolam 31 (ng/mL) Nordiazepam 0.06 Oxycodone 0.08 Cocaine 0.03 Benzoylecgonine 0.3 Ecgonine methyl ester 0.06	Mixed-drug toxicity
5	Protonitazene (1.6)	Q4 2022	20–29	Ethanol 0.01 g/dL Midazolam 0.08 MDMA 1.2 Cocaine 0.03 Benzoylecgonine 0.5 Ecgonine methyl ester 0.06 Cocaethylene 0.02	Hypoxic brain injury and cardiac arrest with mixed-drug toxicity
6	Protonitazene (0.3)	Q4 2022	30–39	Methylamphetamine 0.09 Benzoylecgonine 0.1 Ecgonine methyl ester 0.01 Diazepam 0.02 MDMA 0.3 Ketamine 0.03 Modafinil 0.03 Pholcodine 0.1	Mixed-drug toxicity
7	Metonitazene (0.4)	Q4 2022	30–39	Ethanol 0.02 g/dL Alprazolam 0.05 Methylamphetamine 0.2 Olanzapine 0.1	Mixed-drug toxicity
8	Protonitazene (1.3)	Q2 2023	40–49	Methylamphetamine 0.4	Bronchopneumonia and mixed-drug toxicity
9	Protonitazene (1.7)	Q2 2023	40–49	GHB 53 Methylamphetamine 0.8 Bromazolam 1 (ng/mL) *N,N*-Dimethylpentylone DET > 50 ng/mL Pentylone DET > 50 ng/mL Dibutylone DET > 0.05 ng/mL	Mixed-drug toxicity
10	Protonitazene (0.7) Metonitazene (33)	Q2 2023	30–39	Morphine 0.1, 6 AM urine DET Codeine 0.04 Desalkylgidazepam 3 (ng/mL) 7-Aminoclonazepam 0.07 Bromazolam DET > 50 ng/mL 7-Aminonitrazepam 0.07 Oxycodone 0.02 Amphetamine 0.06 Dextromethorphan 0.02 THC 23 (ng/mL)	Mixed-drug toxicity
11	Metonitazene (18)	Q2 2023	40–49	Bromazolam 16 (ng/mL) Alprazolam 0.03 Amphetamine 0.1 Cocaine 0.2 Benzoylecgonine 7.1 Ecgonine methyl ester 1.5 Norcocaine 0.005 Desmethylvenlafaxine 0.4	Mixed-drug toxicity with pneumonia
12	Isotonitazene (0.08)	Q2 2023	30–39	Ethanol 0.02 g/dL 6AM 0.06 (morphine 0.4) Codeine 0.08 Methylamphetamine 0.5 Salicylic acid 12	Mixed-drug toxicity
13	Metonitazene (11.3)	Q3 2023	30–39	Bromazolam 0.4 (ng/mL) Diazepam 0.08 Methadone 0.4 Methylamphetamine 0.8	Mixed-drug toxicity
14	Protonitazene (0.6)	Q3 2023	20–29	Bromazolam 14 (ng/mL) Desalkylgidazepam 5 (ng/mL) 8-Aminoclonazolam 29 (ng/mL) 7-Aminoclonazepam 0.3 Nordiazepam 0.06 Alprazolam 0.02 *N*-Cyclohexylmethylone 0.6 (ng/mL) Cocaine 0.01 Benzoylecgonine 1.4 Ecgonine methyl ester 0.1 Lamotrigine 3.4 Propanolol 0.2	Mixed-drug toxicity
15	Protonitazene (0.5)	Q1 2024	40–49	GHB (urine) 56 Methylamphetamine 0.8 MDMA 0.07 Sildenafil 67 (ng/mL)	Mixed-drug toxicity
16	Protonitazene (1.5)	Q1 2024	20–29	Bromazolam 0.4 (ng/mL) Desalkylgidazepam 28 (ng/mL) *N,N*-Dimethylpentylone DET > 30 ng/mL Pentylone DET > 30 ng/mL 2-Fluoro-2-oxo-PCE 3 (ng/mL) 3-Hydroxy PCP 13 (ng/mL) Propanolol 0.01 Metoclopramide 0.07	Mixed-drug toxicity
17	Protonitazene (0.1) *N*-Desethylisotonitazene (4.9) Methylenedioxynitazene (3.7)	Q2 2024	20–29	Desalkylgidazepam DET > 50 ng/mL 2-Fluoro-2-oxo-PCE 0.3 (ng/mL) 3-Hydroxy PCP 9 (ng/mL) Bisoprolol 0.02 Valsartan 0.5	Mixed-drug toxicity with dilated cardiomyopathy
18	Protonitazene (11.6)	Q2 2024	40–49	Methylamphetamine 1.4 Benzoylecgonine 0.2 Ecgonine methyl ester 0.05 THC 59 (ng/mL)	Mixed-drug toxicity
19	Protonitazene (3.5)	Q2 2024	30–39	Methylamphetamine 1.9 Cocaine 0.07 Benzoylecgonine 0.05 Ecgonine methyl ester 0.05 THC 9 (ng/mL)	Mixed-drug toxicity
20	Protonitazene (1.3)	Q2 2024	30–39	Methylamphetamine 0.6 Benzoylecgonine 0.06 Ecgonine methyl ester 0.02	Mixed-drug toxicity
21	Protonitazene (4.4)	Q2 2024	<20	Cocaine 0.02 Benzoylecgonine 0.1 Ecgonine methyl ester 0.04 THC 74 (ng/mL) Cannabidiol 11 (ng/mL)	Mixed-drug toxicity
22	Metonitazene (6.2)	Q1 2025	30–39	Morphine 0.04, 6AM urine DET GHB 83 Methylamphetamine 0.6 Nordiazepam 0.5 Desmethylvenlafaxine 0.5 Olanzapine 0.2	Mixed-drug toxicity
23	Protonitazene (0.4)	Q1 2025	30–39	Ethanol 0.01 g/dL Bromazolam 81 (ng/mL) Cocaine 0.05 Benzoylecgonine 0.9 Ecgonine methyl ester 0.4 Norcocaine 6 (ng/mL)	Mixed-drug toxicity

## Data Availability

The data underlying this article cannot be shared publicly for the privacy of the individuals reported in the study. Further inquiries can be directed to the corresponding author.

## References

[1] Hedegaard H., Miniño A.M., Warner M. (2020). Drug Overdose Deaths in the United States, 1999–2018. NCHS Data Brief.

[2] Skolnick P., Paavola J., Heidbreder C. (2024). Synthetic opioids have disrupted conventional wisdom for treating opioid overdose. Drug Alcohol Depend. Rep..

[3] Jones C.M., Einstein E.B., Compton W.M. (2018). Changes in Synthetic Opioid Involvement in Drug Overdose Deaths in the United States, 2010–2016. JAMA.

[4] UNODC (2019). The growing complexity of the opioid crisis. Global SMART Update.

[5] Montanari E., Madeo G., Pichini S., Busardò F.P., Carlier J. (2022). Acute Intoxications and Fatalities Associated with Benzimidazole Opioid (Nitazene Analog) Use: A Systematic Review. Ther. Drug Monit..

[6] Blanckaert P., Cannaert A., Van Uytfanghe K., Hulpia F., Deconinck E., Van Calenbergh S., Stove C. (2020). Report on a novel emerging class of highly potent benzimidazole NPS opioids: Chemical and in vitro functional characterization of isotonitazene. Drug Test. Anal..

[7] Ahmad F.B., Cisewski J.A., Rossen L.M., Sutton P. (2026). Provisional Drug Overdose Death Counts.

[8] Baumann M.H., Tocco G., Papsun D.M., Mohr A.L., Fogarty M.F., Krotulski A.J. (2020). U-47700 and its analogs: Non-fentanyl synthetic opioids impacting the recreational drug market. Brain Sci..

[9] Ujváry I., Christie R., Evans-Brown M., Gallegos A., Jorge R., De Morais J., Sedefov R. (2021). DARK Classics in Chemical Neuroscience: Etonitazene and Related Benzimidazoles. ACS Chem. Neurosci..

[10] Hunger A., Kebrle J., Rossi A., Hoffmann K. (1957). Synthesis of basically substituted, analgesically effective benzimidazole derivatives. Experientia.

[11] De Vrieze L.M., Walton S.E., Pottie E., Papsun D., Logan B.K., Krotulski A.J., Stove C.P., Vandeputte M.M. (2024). In vitro structure-activity relationships and forensic case series of emerging 2-benzylbenzimidazole ‘nitazene’ opioids. Arch. Toxicol..

[12] Walton S.E., Krotulski A.J., Logan B.K. (2022). A Forward-Thinking Approach to Addressing the New Synthetic Opioid 2-Benzylbenzimidazole Nitazene Analogs by Liquid Chromatography-Tandem Quadrupole Mass Spectrometry (LC-QQQ-MS). J. Anal. Toxicol..

[13] Vandeputte M.M., Van Uytfanghe K., Layle N.K., St. Germaine D.M., Iula D.M., Stove C.P. (2021). Synthesis, Chemical Characterization, and μ-Opioid Receptor Activity Assessment of the Emerging Group of “nitazene” 2-Benzylbenzimidazole Synthetic Opioids. ACS Chem. Neurosci..

[14] Schumann J.L., Syrjanen R., Alford K., Mashetty S., Castle J.W., Rotella J., Maplesden J., Greene S.L. (2023). Intoxications in an Australian Emergency Department Involving ‘Nitazene’ Benzylbenzimidazole Synthetic Opioids (Etodesnitazene, Butonitazene and Protonitazene). J. Anal. Toxicol..

[15] Pelletier D.E., Andrew T.A. (2017). Common Findings and Predictive Measures of Opioid Overdoses. Acad. Forensic Pathol..

[16] Kozell L.B., Eshleman A.J., Wolfrum K.M., Swanson T.L., Schutzer K.A., Schutzer W.E., Abbas A.I. (2025). Pharmacology of newly identified nitazene variants reveals structural determinants of affinity, potency, selectivity for mu opioid receptors. Neuropharmacology.

[17] Evans-Brown M., Ujváry I., Morais J.D., Christie R., Almeida A., Jorge R., Gallegos A., Sedefov R. (2020). EMCDDA Technical Report on the New Psychoactive Substance N,N-Diethyl-2-[[4-(1-Methylethoxy)Phenyl]Methyl]-5-Nitro-1H-Benzimidazole-1-Ethanamine (Isotonitazene).

[18] Mueller F., Bogdal C., Pfeiffer B., Andrello L., Ceschi A., Thomas A., Grata E. (2021). Isotonitazene: Fatal intoxication in three cases involving this unreported novel psychoactive substance in Switzerland. Forensic Sci. Int..

[19] Krotulski A.J., Logan B. (2019). Isotonitazene (Analytical Toxicology Report).

[20] Krotulski A.J., Papsun D.M., Walton S.E., Logan B.K. (2021). Metonitazene in the United States—Forensic toxicology assessment of a potent new synthetic opioid using liquid chromatography mass spectrometry. Drug Test. Anal..

[21] Vandeputte M.M., Krotulski A.J., Walther D., Glatfelter G.C., Papsun D., Walton S.E., Logan B.K., Baumann M.H., Stove C.P. (2022). Pharmacological evaluation and forensic case series of N-pyrrolidino etonitazene (etonitazepyne), a newly emerging 2-benzylbenzimidazole ‘nitazene’ synthetic opioid. Arch. Toxicol..

[22] Di Trana A., La Maida N., Froldi R., Scendoni R., Busardò F.P., Pichini S. (2023). The new synthetic benzimidazole opioid etonitazepipne: An emerging fatal harm and a challenge for laboratory medicine. Clin. Chem. Lab. Med..

[23] Shover C.L., Falasinnu T.O., Freedman R.B., Humphreys K. (2021). Emerging Characteristics of Isotonitazene-Involved Overdose Deaths: A Case-Control Study. J. Addict. Med..

[24] Syrjanen R., Schumann J.L., Castle J.W., Sharp L., Griffiths A., Blakey K., Dutch M., Maplesden J., Greene S.L. (2024). Protonitazene detection in two cases of opioid toxicity following the use of tetrahydrocannabinol vape products in Australia. Clin. Toxicol..

[25] Krotulski A.J., Shinefeld J., da Silva D.T., Mohr A.L.A., DeBord J., Walton S.E., Logan B.K. (2023). New Potent Synthetic Opioid-N-Desethyl Isotonitazene-Proliferating Among Recreational Drug Supply in USA.

[26] UNODC UNODC Early Warning Advisory on New Psychoactive Substances. https://www.unodc.org/LSS/Page/NPS/DataVisualisations.

[27] Krotulski A.J., Walton S.E., De Bord J.S., Mohr A.L.A., Logan B.K. (2024). NPS Opioids in the United States Trend Report Q4 2024.

[28] Walton S.E., Garrett R., Denn M.T., Quinter A.D., DeBord J.S., Logan B.K., Krotulski A.J. (2024). Methylenedioxynitazene—NPS Discovery New Drug Monograph.

[29] Partridge E., Stockham P., Kenneally M., Luong A., Kostakis C., Alfred S. (2025). A cluster of multi-drug intoxications involving xylazine, benzimidazole opioids (nitazenes) and novel benzodiazepines in South Australia. EMA Emerg. Med. Australas..

[30] Schumann J.L., Dwyer J., Brown J.A., Jauncey M., Roxburgh A. (2026). Identification of nitazene-related deaths in Australia: How do we make it accurate and timely?. Drug Alcohol Rev..

[31] Protonitazene Sold as Ketamine. https://www.health.vic.gov.au/drug-alerts/yellow-powder-containing-protonitazene-may-be-sold-as-ketamine.

[32] Metonitazene Sold as Cocaine. https://www.health.vic.gov.au/drug-alerts/metonitazene-mis-sold-as-cocaine.

[33] Protonitazene Sold as ‘3C-P’. https://www.health.vic.gov.au/drug-alerts/protonitazene-sold-as-3c-p.

[34] Di Rago M., Pantatan S., Hargreaves M., Wong K., Mantinieks D., Kotsos A., Glowacki L., Drummer O.H., Gerostamoulos D. (2021). High Throughput Detection of 327 Drugs in Blood by LC-MS-MS with Automated Data Processing. J. Anal. Toxicol..

[35] Castle J.W., Syrjanen R., Di Rago M., Schumann J.L., Greene S.L., Glowacki L.L., Gerostamoulos D. (2024). Identification of clobromazolam in Australian emergency department intoxications using data-independent high-resolution mass spectrometry and the HighResNPS.com database. J. Anal. Toxicol..

[36] Krotulski A.J., Papsun D.M., Kacinko S.L., Logan B.K. (2021). Isotonitazene Quantitation and Metabolite Discovery in Authentic Forensic Casework. J. Anal. Toxicol..

[37] Pucci M., Singh Jutley G., Looms J., Ford L. (2024). N-desethyl isotonitazene detected in polydrug users admitted to hospital in Birmingham, United Kingdom. Clin. Toxicol..

[38] Becker J.B., McClellan M.L., Reed B.G. (2017). Sex differences, gender and addiction. J. Neurosci. Res..

[39] Pucci M., Hudson S., Hill S.L., Thomas S.H.L. (2022). Severe toxicity involving N-pyrrolidino etonitazene in the United Kingdom-a case report. Clin. Toxicol..

[40] Pardi J., Ford S., Cooper G. (2023). Validation of an analytical method for quantitation of metonitazene and isotonitazene in plasma, blood, urine, liver and brain and application to authentic postmortem casework in New York City. J. Anal. Toxicol..

[41] Krotulski A.J., Papsun D.M., Walton S.E., Logan B.K. (2021). New Synthetic Opioid Protonitazene Increasing in Prevalence as “Nitazenes” Gain Traction Across the United States and Canada.

[42] Gerostamoulos D., Elliott S., Walls H.C., Peters F.T., Lynch M., Drummer O.H. (2016). To Measure or Not to Measure? That is the NPS Question. J. Anal. Toxicol..

[43] Parks C., Maskell P.D., McKeown D.A., Couchman L. (2024). Identification of 5-aminometonitazene and 5-acetamidometonitazene in a postmortem case: Are nitro-nitazenes unstable?. J. Anal. Toxicol..

[44] Bendjilali-Sabiani J.J., Eiden C., Lestienne M., Cherki S., Gautre D., Van den Broek T., Mathieu O., Peyrière H. (2024). Isotonitazene, a synthetic opioid from an emerging family: The nitazenes. Therapie.

[45] Bendjilali-Sabiani J.J., Eiden C., Lossois M., Martrille L., Peyriere H., Mathieu O. (2026). Postmortem distribution of isotonitazene and its three metabolites in the first lethal case observed in France. Forensic Sci. Int..

[46] Menéndez-Quintanal L.M., Matey J.M., del Fresno González V., Bravo Serrano B., Hernández-Díaz F.J., Zapata F., Montalvo G., García-Ruiz C. (2024). The State of the Art in Post-Mortem Redistribution and Stability of New Psychoactive Substances in Fatal Cases: A Review of the Literature. Psychoactives.

[47] Chen S., Moore R., Hudson S., Copeland C. (2025). Mortality Associated with Nitazenes May Be Underestimated Due to Post-Mortem Degradation. Emerg. Trends Drugs Addict. Health.

[48] Brunetti P., Giorgetti R., Tagliabracci A., Huestis M.A., Busardò F.P. (2021). Designer benzodiazepines: A review of toxicology and public health risks. Pharmaceuticals.

[49] Silva-Torres L.A., Mozayani A. (2024). Xylazine abuse, the growing risk: A review of its effects, upsurge use and associated fatalities in the USA and Puerto Rico. J. Forensic Leg. Med..

[50] Blakey K., Thompson A., Matheson A., Griffiths A. (2022). What’s in fake ‘Xanax’?: A dosage survey of designer benzodiazepines in counterfeit pharmaceutical tablets. Drug Test. Anal..

[51] Mérette S.A.M., Kim S., Davis M.D., Shapiro A.M. (2023). Desalkylgidazepam blood concentrations in 63 forensic investigation cases. J. Anal. Toxicol..

[52] Alhosan N., Cavallo D., Santiago M., Kelly E., Henderson G. (2025). Slow dissociation kinetics of fentanyls and nitazenes correlates with reduced sensitivity to naloxone reversal at the μ-opioid receptor. Br. J. Pharmacol..

[53] Stangeland M., Dale O., Skulberg A.K. (2025). Nitazenes: Review of comparative pharmacology and antagonist action. Clin. Toxicol..

[54] Dahan A., Franko T.S., Carroll J.W., Craig D.S., Crow C., Galinkin J.L., Garrity J.C., Peterson J., Rausch D.B. (2024). Fact vs. fiction: Naloxone in the treatment of opioid-induced respiratory depression in the current era of synthetic opioids. Front. Public Health.

[55] Roberts D.M., Tisdell B., Sajeev M.F., Jiranantakan T., Harvey C., Brown J.A. (2025). Clinical Experiences with the Nitazene Class of Synthetic Opioids: A Cohort Study. Ann. Emerg. Med..

[56] World Health Organisation (2024). Critical Review Report—N-Desethyl-Isotonitazene.

[57] Walton S., Papsun D., Shoff E., Ellefsen K., Krotulski A. (2025). New and Emerging “Nitazene” Analogues Appearing in Medicolegal Death Investigations: N: -Pyrrolidino Protonitazene and: N: -Desethyl Isotonitazene. Am. J. Forensic Med. Pathol..

